# Task rules, working memory, and fluid intelligence

**DOI:** 10.3758/s13423-012-0225-y

**Published:** 2012-07-18

**Authors:** John Duncan, Moritz Schramm, Russell Thompson, Iroise Dumontheil

**Affiliations:** 1 MRC Cognition and Brain Sciences Unit, 15 Chaucer Road, Cambridge, CB2 7EF UK; 2 Department of Experimental Psychology, University of Cambridge, Cambridge, UK; 3 Institute of Cognitive Neuroscience, University College London, London, UK

**Keywords:** Attention, Executive control, Working memory, Individual differences, Memory capacity

## Abstract

Many varieties of working memory have been linked to fluid intelligence. In Duncan et al. (Journal of Experimental Psychology:General 137:131–148, 2008), we described limited working memory for new task rules: When rules are complex, some may fail in their control of behavior, though they are often still available for explicit recall. Unlike other kinds of working memory, load is determined in this case not by real-time performance demands, but by the total complexity of the task instructions. Here, we show that the correlation with fluid intelligence is stronger for this aspect of working memory than for several other, more traditional varieties—including simple and complex spans and a test of visual short-term memory. Any task, we propose, requires construction of a mental control program that aids in segregating and assembling multiple task parts and their controlling rules. Fluid intelligence is linked closely to the efficiency of constructing such programs, especially when behavior is complex and novel.

Tests of “fluid intelligence,” such as Raven’s Progressive Matrices (Raven, Court, & Raven, 1988) and Cattell’s Culture Fair (Institute for Personality and Ability Testing, 1973), are important for their broad ability to predict success in many different kinds of cognitive activity, from laboratory tests to educational and work achievements. Typically, fluid-intelligence tests involve novel reasoning, using geometrical, verbal, or other materials (Marshalek, Lohman, & Snow, 1983). A large research literature has investigated what basic cognitive mechanisms are measured in tests of this sort. In particular, strong links have been suggested between fluid intelligence and working memory (Ackerman, Beier, & Boyle, 2005; Kane & Engle, 2002; Kyllonen & Christal, 1990).

Working memory, however, is a complex concept. It is often proposed that working memory can be fractionated into distinct components, including somewhat separate short-term stores for materials of different kinds (Baddeley, 1986), as well as distinct processing control functions, such as resistance to interference (Kane & Engle, 2002) and cognitive updating (Miyake et al., 2000). Not surprisingly, correlations with fluid intelligence vary widely from one test of working memory to another (Ackerman et al., 2005).

Previously, we have investigated a form of working memory concerning the learning and use of new task rules. The ability to follow new task rules shows substantial individual differences (Duncan et al., 2008). In some cases, failures take a striking form that we have called “goal neglect” (Duncan, Emslie, Williams, Johnson, & Freer, 1996): Though rules can be explicitly recalled, they exert no apparent control over behavior. Critically, neglect increases with the task complexity—that is, the number of rules that the task requires (Duncan et al., 1996; Duncan et al., 2008). This limit, however, seems rather different from capacity limits that have been found in many, more traditional aspects of working memory. In particular, neglect concerns not the processing requirements of an individual trial, but rather the total complexity of the whole set of rules described in the initial instructions. Neglect of a given rule increases with the number of other rules described, even if participants know that, for a given block of trials, these other rules will not be required and can be ignored (Duncan et al., 2008).

Any multistep behavior must be controlled by some internal mental program defining and focusing on separate task parts. At each stage of such a program, relevant information and operations must be assembled, and irrelevant material disregarded, creating an organized series of distinct attentional episodes (Duncan, 2010a). We proposed that, when task instructions are received, they must be transformed into a new control program of this sort (cf. Anderson, 1983; Fitts & Posner, 1967) that separates and defines the distinct task rules as well as conditions for their implementation. This calls for a form of working memory sufficiently large and durable to encompass the complex and multifaceted information that task instructions will typically contain. Neglect occurs when the use of this working memory fails, so that parts of the task control program fail to be implemented.

Our previous results suggested strong correlations between such task-rule working memory failures and fluid intelligence. For simple forms of goal neglect, the correlation with a standard fluid-reasoning task can approach .6 (Duncan et al., 2008). Limitations in the construction of a mental control program, we suggest, are especially well captured in tests of rule working memory when a new, complex program must be built, as well as in standard tests of fluid intelligence, which typically require that a novel series of mental operations be assembled for each new problem (Duncan et al., 2008). As all tasks require some task control program, however, variable ability to produce such programs would help explain universal positive correlations between fluid intelligence and other tasks. This proposal has much in common with other suggestions that have linked fluid intelligence and executive control (e.g., Kane & Engle, 2002; Marshalek et al., 1983; Wilhelm & Oberauer, 2006).

Goal neglect—that is, behavior in violation of task rules, even though these are explicitly understood—is a characteristic failure of patients with major frontal lobe damage (Luria, 1966). In functional brain-imaging studies, fluid reasoning is associated with a characteristic pattern of frontal and parietal activity, incorporating major foci in the inferior frontal sulcus, the anterior insula/frontal operculum, the dorsal anterior cingulate/presupplementary motor area, and the intraparietal sulcus (Bishop, Fossella, Croucher, & Duncan, 2008; Duncan et al., 2000; Prabhakaran, Smith, Desmond, Glover, & Gabrieli, 1997). Lesions within this same set of frontal and parietal regions are selectively associated with fluid-reasoning deficits (Woolgar et al., 2010). The same regions show a pattern of increasing sustained activity as new task instructions are received (Dumontheil, Thompson, & Duncan, 2011). In corresponding regions of the monkey brain, neurons show highly flexible response properties, selectively coding the specific information required at each step of current behavior (Duncan, 2001). Such results are strongly consistent with the requirements of a flexible cognitive control structure, assembling the specific content of each successive step into a current mental program (Duncan, 2010b).

At first sight, working memory for task rules may show an especially close link to fluid intelligence, with weaker fluid intelligence correlations for other working memory tasks. For example, a large review has suggested average correlations in the .2 range between fluid intelligence and simple tests of short-term memory, such as digit span, and only slightly higher values for more complex tasks that mix immediate processing and storage (Ackerman et al., 2005). To compare absolute correlations across studies, however, is hard, since such factors as the ability ranges of participants may not be comparable. Here, we directly compared working memory for new task rules with a variety of more traditional varieties of working memory, including digit span, spatial span, visual short-term memory (VSTM), and a more complex span task that mixed processing and storage.

A variety of tasks can be used to assess working memory for task rules. Here we used two tasks from a recent neuroimaging study in which brain activity was examined as task instructions were received (Dumontheil et al., 2011). In line with our previous work, performance in these tasks is sensitive to the total complexity of the task instructions, irrespective of the set of rules actually operative in the current task block (Dumontheil et al., 2011; Duncan et al., 2008). For a range of similar tasks, previous data from a small group of participants suggested a correlation with fluid intelligence in the region of .6 (Dumontheil et al., 2011). In some cases (Dumontheil & Duncan, 2008, unpublished data; see also data of current study), performance failures take the form of frank goal neglect, with task rules remembered but neglected in behavior.

## Method

### Participants

For optimal correlational data, we sought a broad distribution of fluid-intelligence scores, approximately representative of variability in the normal population. On the basis of prior testing with the Culture Fair test, Scale 2, Form A (Institute for Personality and Ability Testing, 1973), we selected a group of 88 participants ranging in age from 19 to 65 (median 48) years. Their mean Culture Fair IQ was 103, *SD* = 14, range 76–139. The group was selected to ensure that Culture Fair IQ was approximately independent of age, with mean IQs of 103 in both younger and older participants (median split on age).^1^ All participants were paid for their time.

### Testing session

The tasks described here were administered with a number of others in a test session approximately 90 min in total duration. Tasks were administered in the fixed order in which they are described below. For the computerized tests, stimuli were presented on a standard LCD monitor with a resolution of 1,024 × 768 pixels.

### Rule working memory

**Fig. 1:**
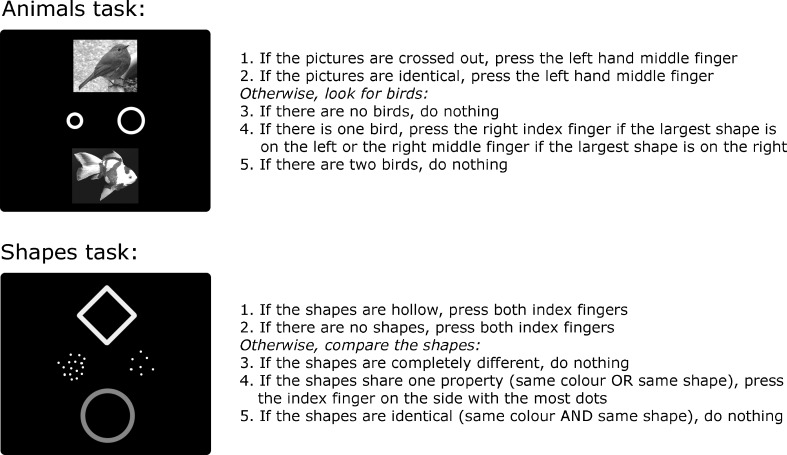
Example stimuli (left) and the five task rules (right) for the animals and shapes tasks. The actual experimental stimuli were presented in color. Rules are numbered in the order of their presentation in the task instructions

Each participant completed two versions of the rule working memory task, one termed “animals” and the other “shapes” (Fig. 1). In each task, five rules (Fig. 1) determined the correct responses to different displays. Each display appeared for 2 s, followed by a 300-ms blank screen before onset of the next display. Responses were made on a standard computer keyboard using the index and middle fingers of the two hands. Participants were encouraged to respond to each stimulus before the next was presented, and responses were accepted up to this point.

Each task began with a series of instruction screens, each presented for 30 s. The first screen contained an overview of the stimuli and responses. Each of the five following screens gave a verbal description of one of the rules (Rules 1–5, in the order shown in Fig. 1), an example of a stimulus display in which that rule would be applicable, and a schematic representation of the response. After viewing all six screens, participants’ memory for the rules was assessed by asking them how they would respond to each stimulus condition. If any incorrect answers were given, participants were corrected and questioned on each rule again. This procedure continued until all of their answers were correct. Participants then completed a practice block containing 16 trials, followed by two experimental blocks, each containing 20 trials. After completing both experimental blocks, rule memory was assessed again. In contrast to the assessment conducted prior to the task, each rule was now probed only once.

For the animals task (Fig. 1), displays were constructed using a wide variety of full-color animal photographs. Each picture subtended approximately 4 deg of visual angle, offset vertically from screen center by approximately ±2.5 deg. The symbols presented between the two animal pictures (Fig. 1) were either two squares or two circles, offset horizontally by approximately ±1.25 deg. The two symbols differed in size, with the larger one always at least 25% greater in height/width than the smaller one (individual shape sizes varied from 0.5 to 1.5 deg, edge to edge).

For the shapes task (Fig. 1), the shapes were circles, squares, diamonds, triangles, hearts, stars, or plus signs, presented in white, blue, red, green, cyan, purple, or yellow. The size of all shapes was 3 deg. Of the two clusters of dots (Fig. 1), one contained at least 50% more dots than the other (individual cluster sizes varied from 2 to 23 dots). The overall display layout matched that in the animals task.

To score rule working memory, each task was regarded as a list of five rules to be stored/used. In each task, the mean percentage of correct responses was calculated for each of the five separate trial types (rules). The net rule working memory score was the mean of these values across the total of ten rules employed.

### Digit span

On each trial, the experimenter read a list of numbers, which the participant was immediately to repeat back in the same order. The test used the materials and procedures from the WAIS-III (Wechsler, 1997). The sequence lengths ranged from 2 to 9 items, each presented twice; the score was the total number of digits correctly recalled. Administration was discontinued if a participant made an error in both presentations of any particular sequence length.

### Spatial span

This test used a plastic board onto which were fixed ten cubes. On each trial, the experimenter touched a series of cubes, which the participant was then to touch in the same order. The test used the materials and procedures from the WAIS-R NI (Kaplan, Fein, Morris, & Delis, 1991). Sequence lengths ranged from 2 to 8 items, each presented twice; the score was the total number of positions correctly recalled. Administration was discontinued if a participant made errors in both presentations of any particular sequence length.

### Operation span

For the operation span test (Engle, Tuholski, Laughlin, & Conway, 1999; Kane et al., 2004), working memory items were interleaved with mental arithmetic. For each item, participants were presented with a visual display that included a mathematical equation followed by a word—for instance, (4 × 2) – 3 = 5 :: STORE. Participants were asked to indicate verbally whether or not the equation was correct and then to repeat the word out loud, after which the experimenter presented the next item. After a series of items, participants were asked to recall all of the words presented during this series, in any order. Series (memory lists) contained 2, 3, 4, 5, or 6 items, and participants completed three repetitions of each list length. All participants were presented with the different list lengths in the same random order.

All displays were presented in white, 14-point Arial font on a black background. All of the equations contained two operations: a division or multiplication (presented in parentheses) followed by either an addition or a subtraction. All of the values used on the left side of each equation were single-digit numbers from 1 to 9. The equations were given with incorrect answers (deviating from the correct answer by either 1 or 2) on half of the trials. The words were monosyllabic concrete nouns of length 4–6 characters, with Kučera–Francis (1967) written frequency values ranging from 45 to 75 and concreteness ratings from 365 to 670. The score was the total number of words recalled correctly.

### Visual short-term memory

In the VSTM task (Cowan et al., 2005), each trial began with a fixation cross presented in the center of the screen for 1,000 ms. Participants were then presented with an array of colored dots for 150 ms. After a delay of 1,200 ms, a single probe dot was presented for 1,750 ms, and participants were asked to indicate whether the probe was the same color as the dot that had appeared in the same position in the original array. After a brief practice, participants completed 180 experimental trials, with 36 repetitions of five different array sizes (1, 2, 4, 6, and 8 dots) presented in random order. Within each set size, 18 trials contained probes whose color matched the dot in the original array, while the remaining half included nonmatching probes. The order of matching and nonmatching probes was randomized. Nonmatching probes featured either a color that had been presented in a different position in the original array or a novel color. Dots were presented in random positions within a notional 3 × 3 grid aligned to the center of the screen. Individual dots had a diameter of approximately 0.5 deg of visual angle, and the edges of neighboring dots were separated by approximately 0.4 deg, so that the maximum extent of the whole display was approximately 2.3 deg. In total, ten different colors were available, and the colors used on any particular trial were chosen at random from this set. Responses were made on a standard computer keyboard by using the left shift key to indicate that the color was the same as in the original array and the right shift key to indicate that the color was different. The score was the percentage of correct responses across all array sizes; this produced a slightly higher reliability and IQ correlation than did a score based only on the larger array sizes (4–8 dots).

## Results

Descriptive statistics for each measure are shown in Table 1. Correlations (Pearson’s *r*) between all measures are shown in Table 2, with reliabilities for the working memory scores on the diagonal. Reliabilities were based on the Spearman–Brown formula, calculated for rule working memory using the average correlation between scores on the ten individual rules, and for the other tests using either a standard split into halves (digit span, spatial span, VSTM) or a split into thirds (operation span; each third with one list of each length).

**Table 1:** Descriptive statistics

Test	Score	Mean (*SD*)
Culture Fair	IQ	102.6 (13.5)
Rule	Percent correct	56.2 (21.3)
Digit	Total correct	62.0 (12.2)
Spatial	Total correct	43.6 (8.4)
Operation	Total correct	43.2 (7.0)
VSTM	Percent correct	76.1 (7.2)

**Table 2:** Correlations between all measures

	Culture Fair	Rule	Digit	Spatial	Operation	VTSM
Rule	.57	.79				
Digit	.38	.26	.79			
Spatial	.45	.42	.16	.56		
Operation	.35	.26	.53	.17	.88	
VSTM	.36	.32	.12	.32	.20	.88

**Fig. 2:**
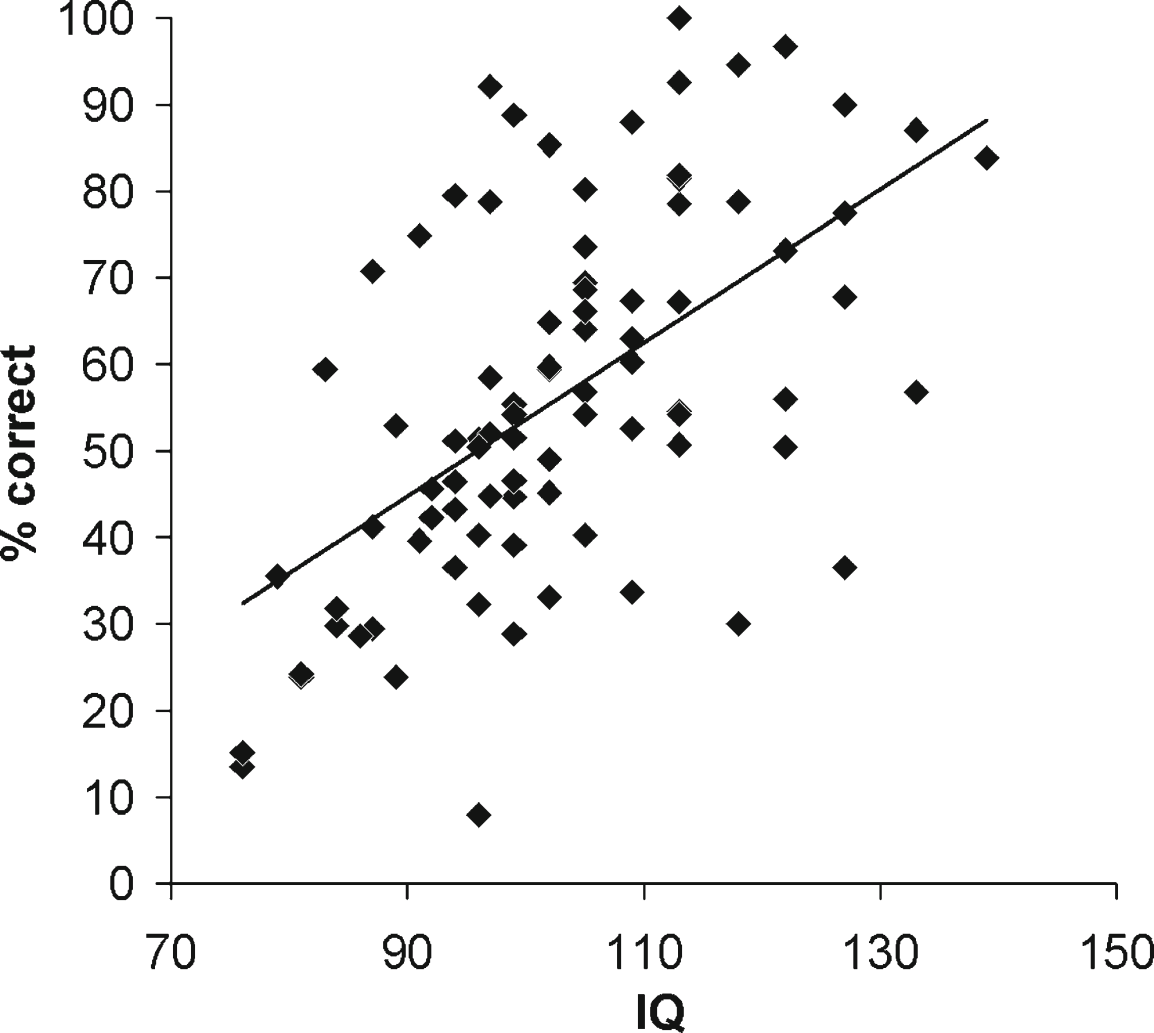
Scatterplot and the best-fitting regression line for the relation between rule working memory score (*y*-axis: mean percentage correct across all five rules and two tasks) and Culture Fair IQ. Each point corresponds to 1 participant

Of the five types of working memory assessed, rule working memory showed the highest correlation (.57) with Culture Fair IQ. As is shown in Fig. 2, the link of rule working memory to Culture Fair score held across the IQ range. For other tests, the Culture Fair correlations ranged from .35 (operation span) to .45 (spatial span). The correlation with Culture Fair was significantly higher for rule working memory than for digit span [Williams test, one-tailed, *t*(83) = 1.69, *p* < .05], operation span [*t*(83) = 1.98, *p* < .05], or VSTM [*t*(79) = 1.84, *p* < .05]; for spatial span, the difference was not significant [*t*(83) = 1.23, *p* = .11]. (The variable *df* values in these tests reflect occasional missing scores.) From the published reliability of the Culture Fair (.87; Institute for Personality and Ability Testing, 1973) and the reliability of .79 of rule working memory (Table 2), correction for attenuation would raise the correlation between these two to .69.

To define a “general intelligence” factor, a well-established alternative to fluid-intelligence tests is principal-components analysis, where the first principal component accounts for much of the general tendency to positive correlations in a diverse battery of tests (Nunnally, 1978). In our data, this approach led to similar conclusions. Using the complete battery of six tests, the first principal component accounted for 41% of the variance. As expected, the strongest loading on this first principal component was obtained for Culture Fair (.79), followed by rule working memory (.71), spatial span (.65), operation span and VSTM (both .56), and digit span (.52).

In each rule working memory task, performance on the individual rules varied widely. For each rule in each task and participant, we used an arbitrary criterion of <25% correct (cf. Duncan et al., 2008) to define frank rule neglect. Combined across rules, tasks, and participants, the mean rate of neglect was 28%. Averaged across the two tasks, Rule 2 (Fig. 1) was most frequently neglected (mean neglect rate = 42%), and Rule 5 was least frequently neglected (19%). Even when a rule was neglected, this was usually (65% of neglect cases) associated with correct rule recall at the end of the task. As in previous studies, the results confirm that neglect often occurred with intact explicit memory for the task rules. To confirm that explicit memory failure did not drive the Culture Fair correlations, the rule working memory score for each participant was recalculated after excluding rules not finally described correctly. This left the correlation with Culture Fair unchanged (.57).

Although age varied widely in our sample, this variation had little effect on Culture Fair correlations. Partialing out age slightly increased the Culture Fair correlation (to .59) for rule working memory, and for the other working memory tests, it changed the Culture Fair correlation by a maximum of .02.

## Discussion

These results confirm a strong link between fluid intelligence and working memory for task rules. At .57, the correlation between Culture Fair and rule working memory was significantly higher than the equivalent correlations for digit span, operation span, and VSTM, and it was numerically higher than the correlation for spatial span. We propose that, in all tasks, a mental control program must implement an integrated series of attentional episodes defining the separate parts or stages that the task contains (Duncan, 2010a). If fluid intelligence is linked closely to the ability to construct such programs, it may be measured especially well when new task programs are assembled, defining separate task parts and the conditions that elicit them.

Given this proposal, an open question is how best to interpret the correlation of fluid intelligence with other working memory tests, including those examined in the present study. Many previous studies have reported positive correlations between fluid intelligence and different varieties of working memory; on average these have been quite modest (Ackerman et al., 2005), but in individual studies they were sometimes substantial (e.g., Fukuda, Vogel, Mayr, & Awh, 2010). One possibility is that all of these correlations reflect the same basic function of controlling any task by assembly of an appropriate control program or structure of attentional episodes. Digit span, for example, requires phonological working memory for the actual digits presented on a single trial, but at the same time, a broader mental control program must ensure, for example, that attention is paid correctly to the input as digits are presented, and then switched smoothly to output as they are repeated. The importance of the broader control representation is suggested by correlations between fluid intelligence and all kinds of tasks, even those not overtly concerned with short-term information maintenance. It remains possible, however, that fluid intelligence links to multiple separate aspects of working memory, including maintenance of a specific stimulus, as well as broader task control.

In this context, an intriguing finding is the comparatively strong correlation between fluid intelligence and spatial span. In many kinds of task, frontoparietal activity—resembling that linked to fluid-reasoning tasks—increases with task difficulty (Duncan & Owen, 2000). An exception occurs in spatial span, in which activity is increased by the occurrence of organized spatial chunks (Bor, Duncan, Wiseman, & Owen, 2003), while at the same time such chunks improve performance. Although fluid intelligence may be linked to spatial maintenance per se, an alternative is that it is linked to the organizational process of detecting and using novel chunks.

Though any task will show some positive correlation with fluid intelligence (Cattell, 1971; Spearman, 1927), the magnitude of these correlations varies substantially. One critical factor is task complexity, with the lowest correlations generally being associated with simple and repetitive tasks, and higher correlations being associated with tasks combining multiple novel parts (Marshalek et al., 1983; Stankov, 2000). Correspondingly, the best tests of fluid intelligence traditionally require novel problem solving, in which each individual problem requires a new, often complex, set of component parts to be identified and assembled. As the present results show, however, complexity and novelty rather than problem solving per se may be critical in producing high fluid-intelligence correlations. When new task rules are explicitly described, there is no overt element of problem solving. Nevertheless, converting these instructions into an effective task control program is closely linked to fluid-intelligence scores.

When behavior is simple and familiar, the quality of the control program may contribute only modestly to between-person variability. With increased complexity and novelty, however, programs may vary substantially in their effectiveness, increasingly contributing to individual differences. In this respect, the present measures of rule working memory may form a bridge between traditional, relatively simple working memory tasks, with their relatively modest fluid-intelligence correlations, and the full complexity of novel reasoning.

## Footnotes

1 As adult norms for the Culture Fair are not age-corrected, different age groups matched for IQ are also matched in terms of absolute performance.
